# How Russia’s war in Ukraine can change gender studies

**DOI:** 10.3389/fsoc.2023.1220438

**Published:** 2023-11-30

**Authors:** Janet Elise Johnson

**Affiliations:** Brooklyn College (CUNY), Brooklyn, NY, United States

**Keywords:** intersectionality, decolonization, solidarity, Ukraine, postsocialisms, Russia, gender studies

## Abstract

The catastrophe of Russia’s war in Ukraine following on the heels of the COVID-19 pandemic and the Black Lives Matter-inspired protests raises the possibility of marked changes in people’s gendered experiences in Central-Eastern Europe and Eurasia (CEE&E). Drawing upon recent discussions, events, and publications—with particular attention to Ukrainian voices and reflexivity as to my position as a US-based political scientist mostly trained on Russia—I suggest ways that these developments have begun to, can, and should change gender studies. I raise three questions: (1) Does CEE&E still constitute a meaningful geopolitical context—or region—for understanding gender? (2) Who should have authority to speak about gender in CEE&E? and (3) Have the possibilities for solidarities among feminist activists across CEE&E and beyond CEE&E increased or decreased, and why? Considering these questions, I argue that there is still much to be gained from studying gender in CEE&E as a field if we incorporate intersectional and decolonial lenses and especially if we can keep pushing ourselves through the scholarly debates that have constituted the field. Incorporating this part of the world—elided since Soviet collapse—can help gender studies as a whole deepen and reconsider paradigmatic concepts such as intersectionality, colonialism, and solidarity.

## Introduction

Within its first year and a half, Russia’s full-scale war against Ukraine in 2022—marked by intentional brutality and with no justification—has killed hundreds of thousands of people, uprooted and traumatized millions of others, terrorized the nearby countries’ citizens, increased food insecurity in Africa dependent on imported grain, and what seemed to be a post-Cold War settlement. Over the last several decades, feminist scholars have unpacked the ways that modern wars are gendered. In brief, they show that war is constructed around militarized masculinities casting men as warrior protectors and shrinking women into “passive sobbing wartime victims” (and sometimes also “unreal super-heroines”; Enloe, 2023). These stories reinforce existing gendered roles and sexist construction of nations and obfuscate the ways in which men often perpetrate violence against women within their communities. To understand war, feminists around the world have taught us that “it is crucial to stay curious about the full range of women’s gritty wartime lived realities” (Enloe, 1993, 2023).

Russia’s war in Ukraine is gendered in ways that sometimes resemble previous wars and sometimes not. Most men in Ukraine have been militarized or unable to leave the country, but some one-fifth of the military was made up of women, who have been empowered through Ukrainian women’s Invisible Battalion project (Hendrix and Korolchuk, 2022). Russian commanders appear to have condoned or even encouraged rapes during the war, with a “an even clearer pattern…of organized sexual abuse in the detention facilities run by Russian troops, police officers and security forces” (Gall and Boushnak, 2023). But, Ukrainian and international human rights advocates immediately started documenting while also providing concrete and empowering assistance rather than using their narratives for national or international aims (Workshop Feb. 24, 2023).^1^ While Putin has been justifying the war as a way to protect families and children from gender equality and LGBTQ+ rights (Novitskaya et al., 2023), the Ukrainian government—after years of activism, a well-supported petition in support, and gender equality advocate Maryna Bardina advising Zelensky—ratified the Istanbul Convention on gender violence, the fiercest convention for gender justice in the world. Ukrainian society, seeing LGBTQ+ soldiers and building on post-2014 Revolution of Dignity activism, has compelled important conversations about LGBTQ rights, leading to a new law against LGBTQ hate speech and draft legislation to legalize same-sex unions.

This war followed other gendered global developments with impact in Central-Eastern Europe and Eurasia (CEE&E). Fear of COVID-19 and public policy forced many women back into homes which were less likely to be safe from domestic abuse in CEE than in their Western European counterparts because governments remain less responsive; lockdowns also deprived children of childcare and schooling, exacerbating the impossible postsocialist balancing act of most working mothers. Isolation increased at the micro and macro level as borders closed, even for those living in the European Union, and many lived with increased fear and exhaustion, with fewer economic resources as the reverberations of lockdowns hit the economy. Public health mandates energized right-wing populist movements, many of which have anti-feminist and anti-LGBTQ rights politics. In addition, the spring–summer 2020 Black Lives Matters protests in the United States spurred protests against racism in CEE&E, empowering postcolonial and decolonial critiques in the “post-socialist peripheries” (Adriana Zaharijević ECPG, 2022). This wave of protest came soon after the 2016 #Iamnotafraidtosay online flashmob, initiated by Kyiv-based feminist Anastasiya Melnychenko, and the 2017 transnational #MeToo movement started by US Black feminist Tarana Burke.

We scholars of CEE&E have been learning from the collapse of Soviet control how much these global developments do (and do not) change gender (Johnson et al., 2021; Regulska and Włodarczyk, 2022). In this article, I consider whether these last few years, especially the catastrophe of the war, mark a critical juncture in the study of gender in CEE&E, as well as reflect on how this consideration can help change gender studies.

To do so, I begin with my experience as co-editor with Fábián et al. (2022) of *The Routledge Handbook of Gender in Central-Eastern Europe and Eurasia* (hereafter *Handbook*). As evidence of change, I use participant observation to notice the tensions around what is said by, to, and about Ukrainians in various exchanges and academic events in the US and Europe. They include panels and roundtables I attended at the European Conference on Politics & Gender in Ljubljana (ECPG) in July 2022,^2^ and the Association for the Study of Eastern European and Eurasian Studies (ASEEES) in Chicago (and online) in the Fall 2022,^3^ and at the first day of the hybrid conference related to this special issue, “Gender, Civil Society, and Women’s Movements in the Context of Russia’s War on Ukraine” at Rhine-Waal University of Applied Sciences (hereafter Kleve) on January 18, 2023. I also draw upon interactions as co-coordinator with Mara Lazda of the Gender and Transformation in Central-Eastern Europe and Eurasia Workshop (hereafter Workshop) now at the CUNY Graduate Center^4^ and as member of the Association of Women in Slavic Studies (hereafter AWSS). In addition, I pay special attention to some Ukraine-focused sources from the first year: the feminist “Gender in Detail” site;^5^ the special issue of the Kharhkiv Center for Gender Studies (2022); and the weekly series on the war in Ukraine through FRIAS at the University of Freiburg, Germany, in the Spring/Summer of 2022.^6^

This analysis is perhaps premature and surely partial. However, I aver that it is important and useful to do this thinking even under these circumstances, not just because of what is happening to those in Ukraine or because the population of CEE&E is some one-sixth of the world’s, but because the elision of CEE&E into the Global North has left gender studies intellectually impoverished. Taking CEE&E seriously can help feminists wrestle with the travel of the concept of intersectionality beyond its founding in the US with a “intellectual and social justice mission” to center the experiences of Black women (e.g., Dill and Zambrana, 2009). Noting the tendency of scholars to focus on domestic dynamics, feminist scholars of the Global South such as Patil (2013) have argued that colonialism should be considered as one of the interconnected structures of inequality. CEE&E scholar Tlostanova (2010, 2022) has expanded this decolonial thinking to Central Asia, the Caucasus, and Siberia’s Far North, but not much thought has been given within gender studies as to what to do with colonialism that does not justify itself in racial ways. As elaborated below, Russia has long justified its conquest of Ukraine, along with Belarus, by the alleged absence of racial, cultural, and linguistic differences. Thinking about the war also helps bring into view the gritty gendered brutality of 21st century colonialism’s conquest and how to build (or not build) feminist solidarities in a time of war.

## My/our partial decolonization project

This is a decolonizing project for me. I am US-born and -based, of Western European heritage, a feminist political scientist who came to study Russia in the 1990s. Raised by Republican Party-identified parents with an ideological bias against the Soviet Union, my upbringing also committed me to learning about others through close readings of embodied experiences, to expanding my capacity for compassion, and to question all violence. I traveled to the Soviet Union in 1991, with both perspectives, and the trip brought the sexism I had experienced into my awareness.

I had thought that my gendered lens would help me see the fullness of the Russian regime’s capacity for violence, but I was disciplined as a comparative political scientist to take as given state borders and ideologically constructed regions, and that discipline made me underestimate Russia’s imperialist agenda. Similarly, my training came at the height of Western triumphalism that constituted transition theory, which I knew was unjustified given the failures to address gender equality, but it still structured my—and the broader field’s—lack of recognition of how much the Soviet collapse was a potentially decolonization process. Similarly, my interdisciplinary orientation toward regional studies limited my grasp of the full extent of the violence Russia imposed beyond the region, such as in Syria, Libya, and the Democratic Republic of Congo.

Over the last decade, I became postcolonial in my teaching, examining the colonizing projects of both Russia (but also the US and other colonizers) and considering more seriously the experiences of the rest of the region and world with colonialism. In the *Handbook*, my co-editors and I worked hard to incorporate postcolonial and decolonial perspectives (e.g., Gradskova, 2022; Shchurko and Suchland, 2022) as well as de-center Russia by being as comprehensive in covering CEE&E as possible and bringing in Baltic, Belarusian, and Central Asian perspectives on the USSR. I (with my co-editors) argued that Russia and Soviet colonialization should become another important structure in the intersectional analytical matrix of race, class, gender, sexuality, disability, etc., that differentially situates people in profound ways. Since 2022, more scholars of gender in CEE&E have been considering what kind of decolonizing project this is, as Mayhill Fowler recommended (ASEEES 2022), given that the Soviet system included not just mass violence, but also mass participation. Gender scholars (e.g., Ghodsee, 2018) assert that Soviet modernization included emancipatory promises and projects for women. As Hinterhuber and Fuchs (2022) explore in the *Handbook*, the extent of Soviet emancipation has been and should continue to be a central debate for those of us who study gender in CEE&E.

But this war has been a deeper reckoning. As fellow Russianist-political scientist Yoshiko Herrera said at ASEEES (2022), “if you are not reconsidering your life right now, you are not doing your job.” While some of my Western-based colleagues have decided to stop focusing on gender and CEE or on Russia as a result of the fraughtness of all this, I persevere, cautiously. Russianists are important. As Yevgenia Albats said in the same panel, this is “a great tragedy, not just a field of research. We have to study Russia, even if we want to decolonize because this war will have a profound impact on the world.” While Albats claims that Russianists “were so enamored in Putin’s macho-ness” that they missed his 2008 “silent coup,” feminist Russianists were not so insensitive to its violence (e.g., Sperling, 2015; Gradskova, 2020; Johnson et al., 2021) and should, I insist, have more sway in the male-dominated field. However, I know that my feminist study of CEE&E had remained Russia-centric not just in focus, but in perspective. Ukraine has been a “side project,” but now I am trying to centralize it even when I write about Russia. At ASEEES (2022), Milada Anna Vachudova stated that “it’s not bad when Russianists retool to focus on Ukraine—we all retool” which is different from claiming authority as some have done without doing the work. Vachudova specifically called for more work on gender.

As a recovering Russianist during what seems to be the interminable middle of Russia’s war against Ukraine, the project here is partial in both senses of the word. First, while other scholars work to keep the complexity of the global impact in view (e.g., Enloe, 2023), I center feminist scholarly voices on Ukraine and the experiences of Ukrainian (and other Central-Eastern Europeans) women over other decolonial approaches. As Hendl (2022, 63) wrote in the Kharkiv journal, “on an international level, Ukrainian voices and agency are persistently being marginalised and erased.” This article is part of a broader project to include more scholarship from CEE&E, as it is almost completely missing from the reproduction of women’s and gender studies globally, with virtually no scholarship by scholars at institutions in CEE&E in introductory textbooks, encyclopedias, and the most prominent journals outside of CEE (Wöhrer, 2016). Nachescu (2018; see also Marciniak, 2006; Suchland, 2011) explains that transnational feminism has elided what had been called (problematically) the Second World, implicating Eastern Europeans as part of the West/Global North, even as they have been seen as “undesirable Europeans” in Western Europe and the US. At the same time, I recognize that many Central-Eastern Europeans have been privileged by race--what others called “peripheral whiteness” (Safuta, 2018) or “new immigrant whiteness” (Sadowski-Smith, 2018)—and that White Ukrainians elicited unusual sympathy at the onset of the full-scale war. Given this focus and the limits of a journal article, I regret that I do not write more here about the impact of the war on global food insecurity in Africa, which is “gendered even in patriarchal peacetime” (Enloe, 2023, 9), or about the take on the war from feminist scholars in the Global South, whose understanding of Russia’s colonialism is very different.

Second, as feminism is my primary commitment and empiricism my secondary, the project is partial too in its epistemology. As I understand it, many decolonial theorists, such as Tlostanova (2010, xviii-xix), are more post-structural, resulting in privileging colonialism within the hierarchy of oppressions and questioning the very foundations of social science theorizing. At least for now, I see colonialism as one of the many potentially significant intersectional analytical categories, dependent on the empirical context and research question. As I explore below, I also hold that positionality is important to contend with, but I think we can and should try to theorize across place and time. Finally, as a pragmatic feminist, I am committed to possibility of agency within these broader structures; in this instance, I see the possibility and importance of feminist anti-war resistance without and within Russia. But, I recognize my limits: as Tlostanova (2022) points out, peace and conflict studies is rooted in modernity, and indigenous peoples have different ways of knowing which should inform this scholarship.

At the same time, I am inclined toward Narayan’s (1997) feminist postcolonialism, not just focusing on the “cultural riches” of colonized societies or the egregious behaviors of the West, but aiming for a critical analysis that takes on colonialist legacies but also bad governing choices, while paying special attention to scholars of and from the colonized societies but not assuming they all have the same view or that they only have expertise on their own countries. Postcolonial societies and Western postcolonial theories have sometimes valorized misogynist and LGBTQ-phobic “traditions,” empowering religious or male elites’ authority over women’s (and LGBTQ people’s) voices. While Hrycak (2022) makes a powerful argument about the Soviet legacy and Russia’s interference in Ukraine’s institution building and policy response to domestic violence, we cannot assume that Ukraine’s government is blameless when it comes to addressing gender-related issues. As Channell-Justice (2022) shows in her book—analyzing how activists in Ukraine, including feminist ones, worked around and instead of with the state up through the 2014 Revolution of Dignity—Ukraine’s government has been plagued by corruption and ineffectiveness.

From my feminist (partial) decolonizing perspective, I rephrase my research question as the following: how has Russia’s war in Ukraine and the Ukrainian resistance—on top of the recent other global developments and informed by the perspectives of Ukrainians and Ukrainianists, especially Ukrainian feminists—changed the study of gender in CEE&E? In the following, I raise and consider three interrelated questions and suggest my take, acknowledging that they come from my position as a White, US-based Russianist.

## Raising questions and some tentative thoughts

### Does CEE&E (still) constitute a meaningful geopolitical region for understanding gender?

The *Handbook* co-editors and I already had our doubts about the boundaries around what some call the postcommunist region as being a meaningful context for seeing similarities more than differences, the usual justification for area studies. In a part of the world divided by different religions, language groups, and empires with different gendered norms and rules, the shared experience of state socialism with its particular approach to women’s emancipation—in the workforce and social policy, though little attention toward gendered violence—had been the justification for it being considered a region in the second half of the twentieth century. But, as the *Handbook* shows, we have learned so much these last few decades about how, even during this period, there were marked differences regarding gender and sexuality. For example, Romania’s totalitarian approach toward women’s reproduction under Ceausescu contrasted with the legalization of abortion elsewhere. The Soviet (and Romanian) repression of homosexuality contrasted with what we have learned was much more liberatory approaches in CEE (Takacs, 2022). Racial lenses complicated this further, with the Roma facing repressive reproductive control and less progressive social policy, suggesting practically no socialist emancipation (Varsa, 2022). Krylova (2022, 47) suggests that the Cold War origins of CEE&E area studies has limited our ability to see some of the gendered dynamics for what they are, missing some “alternative, non-binary, and, yet, heterosexual forms of organization of family, work, self” under state socialism.

In the post-Soviet period, different divisions within CEE&E emerged, especially between those countries allowed to join European and Western institutions, especially the EU which required at least the pretense of gender equality reforms as part of other requirements of democratization and which overall did better at providing economic welfare (Spehar, 2022). While most CEE, especially Baltic, countries brought prominent women into power in the 21st century (Wolchik and Chiva, 2022), most non-Baltic former Soviet states consolidated as hybrid or authoritarian regimes, patronalist in their male-dominated elite networks topped by hegemonic male patron-presidents (Johnson, 2016). It is important to note the backsliding of the previous success stories—Hungary and Poland—in terms of gender justice, LGBTQ-phobia, xenophobia, as well as democracy, engineered by leaders claiming to be “strong men” along the lines of Putin. Elżbieta Korolczuk (ECPG 2022) called it “premature consolidation,” with gendered connotations, and suggested the analytical framework of autocratization instead of backsliding.

In working on the *Handbook*, the approach that challenged the construction of CEE&E as a region the most were postcolonial, but more significantly, decolonial lenses. There were several chapters that empirically explored Russian and Soviet colonialism. Yulia Gradskova (2022), for example, argued that the coercive unveiling campaigns in Central Asia and Caucasus were hardly emancipatory for women there, instead discursively framing Russian women for being “civilized.” Budryte (2022) considered the Soviet coercion in the deportation and incarceration of Baltic women to Labor Camps, fostering state-administered or explicitly condoned gendered violence.

We also invited Tlostanova (2010), author of *Gender Epistemologies and Eurasian Borderlands* (2010), who, along with Suchland (2011), had argued for a decolonial framework using the concept of Eurasia to bring CEE&E into the internationalization of women’s and gender studies and postcolonial theorizing. Tlostonova drafted a chapter for *Handbook*, but when we asked for revisions to clarify some claims and key concepts, including about what she meant by Eurasian borderlands, she withdrew from the project. It seems that we met an epistemological impasse that we could not remedy. While she was doing the work of questioning and deconstructing the concepts, we came from an epistemology that requires some common language to communicate and collaborate with each other. Shchurko and Suchland (2022, 71) contributed a chapter arguing that [w]hile some scholars are unreflective of, or resistant to, anti-imperial frameworks… others have taken the question of postcoloniality as an opportunity for internal, reckoning and reevaluation.” We took these concerns seriously, and the final versions of our editors’ introductions in the *Handbook* avoid simple constructions, eschewing the word “region” for the more complicated acronym CEE&E.

Revisiting these questions in 2022–3 from the perspective of Ukraine raises more decolonial questions. Since 2014 and even more loudly since 2022, feminists in Ukraine have been asserting that Ukraine too was colonized. The colonization of Ukraine, as Galyna Kotliuk (Kleve) argued, has been overlooked because postcolonial theorists often think about colonialism as about overseas vs. “adjacent” territories and most tend to be Marxist; racial differences also tend to be required, while Ukraine’s otherness is obfuscated because of their Whiteness, even their difference from Russians hidden by Russia’s centuries-long elision of Ukraine and Belarus under the so-called “Trinity of Slavic Peoples, of Great, Little, and White Russians.” The uncritical repetition by many on the Left in the West of the refrain that this is a “US/NATO proxy war against Russia”—echoing Russia’s Foreign Minister Sergei Lavrov (Brands, 2022)—suggests how much Russia’s colonialism in Ukraine, have been rendered so invisible that they can appropriate propaganda. Scholarship on Russia by Ukrainianists such as Timothy Snyder (2010) and Yana Prymachenko^7^ shows Russia’s long intentional campaign to sow confusion and distort reality to gain control over the memory and understanding of Ukraine.

The gendered study of CEE&E has participated in this lack of knowing. As Ukrainian feminist Hanna Hrytsenko (2022b) argues, “the women’s issue in the Soviet Union was constructed at the intersection of the claimed emancipation of women (which was indeed partially carried out, especially in the early years of Soviet rule) and specific national policy.” But Alexandra Hrycak (ASEEES 2019) pointed out, feminist scholars who highlight the early Soviet emancipatory project, even when they note the colonialism in the Caucasus and Central Asia, tend to forget the 1921–1923 famine in Ukraine which some scholars label genocide, not just the later Holodomor (1932–33). Similarly, gender studies scholars of CEE&E have not tended to perceive that Russian colonialism played a part in the post-Soviet period (Hrytsenko, 2022b). Given this colonial and genocidal history, many feminists in Ukraine do not see a contradiction between feminism and nationalism or asking for more weapons to defend Ukraine. This is also true in other countries, such as the Baltics, which also face or fear Russia’s war crimes.

With many colleagues (such as at the ASEEES 2022 panel, “The Gender of War: Central and Eastern European Anti-Gender Crusades”), I suggest that one shared experience that continues to define this part of the world for gendered analysis in 2023 is the particular politics of anti-genderism. As Adriana Zaharijević (ECPG 2022) explained: “I agree with Maria Bucur who recently claimed that ‘gender is becoming a point of interest and fostering connections, it seems, more vigorously among opponents of gender mainstreaming, feminist politics, and LGBTQ rights, than among activists and scholars of gender studies.’” Anti-genderism is a global movement, with strong influence from US evangelicals and the Vatican, but regimes such as Poland and Hungary openly embraced the movement’s claims—casting this move as an anti-imperial reaction against Soviet policy that claimed emancipate women—while other regimes deferred to anti-genderism to justify their failure to ratify Istanbul convention (Graff, 2022). But over the next decade, with Russia’s passing its anti-“gay propaganda law,” the Kremlin recast the movement as anti-Western. As Elżbieta Korolczuk (ECPG 2022) argued, “Putin’s Russia has become one of the key promoters of anti-gender movements to destabilize the EU and democracy by supporting extremist groups.” She explained that anti-genderism is not ideology in itself, but aa “counterknowledge that is blurring the boundaries of what gender studies is.” The techniques of the anti-gender movement, also now shaped with Russia’s influence, about needing to “protect *our* children” and doing the opposite, are similar to the broader disinformation campaigns identified by Snyder (2010) and Prymachenko.

Some Ukrainian feminists and even the Ukrainian government see anti-genderism as part of Russia’s decade-plus re-colonizing conquest, with the evidence of war rape, as the most extreme, violent version of anti-genderism (Hrytsenko, 2022b). Lukasz Niparko (ASEEES 2022) declared that “anti-genderism materialized in Russia’s war in Ukraine” in its deadly form. In the years leading up to Russia’s full-scale war and in the aftermath of the women-led protests in 2020, Belarus upped its anti-genderism, following Russia’s lead, codifying in its constitution norms of marriage as between men and women and introducing more homophobia while simultaneously getting rid of the private schools that were teaching Belarussian (Vera Beloshitzkaya ASEEES 2022). While Olena Strelnyk (Kleve) suggests that Ukraine’s post 2022 progressive moves on gender and LGBT issues were designed to bolster Ukraine’s bid for EU candidacy, Galyna Kotliuk (Kleve) argued that as the war in Ukraine is an anti-colonial war, and the positive changes toward gender are one way to decolonize Ukraine. While it is diverse in many important ways, CEE&E remains united by Russian and Soviet gendered colonializing projects with their particular dynamics, such as the colonizer presenting itself as decolonizing, and, in some cases, tactically eliding rather than exaggerating racial differences.

This discussion shows that feminist study of CEE&E has participated in colonial unknowing, but also the decolonial knowing especially since the 2022 full-scale invasion. As elaborated in the framework developed by Regulska and Włodarczyk (2022), scholars can move beyond the Cold War framing of binaries and borders while being empirically informed and connecting the study of the complex and embodied empirical realities of these varying places in CEE&E. Intersectional postcolonial analytical lenses are highlighting the very different degrees of Russian/Soviet coercion involved in state socialist women’s emancipation and revealing the degree to which it was imposed “from above” and built upon and reinforced colonial and/or racist assumptions (e.g., Gradskova, 2022). Feminist-informed postcolonial approaches are bringing clarity to the differentially experienced but still imperial ambitions and role of post-Soviet Russia (Tlostanova, 2010; Shchurko and Suchland, 2022). With more epistemological nuance evident in recent intersectional feminist theorizing, I think that we can hold the complexity of the colonial, transnational, racialized, and gendered operations of multiple axes of power in and around CEE&E if we keep the conversation multivocal. I think that this is not only my own approach but one that those from the region also considered important. For example, Ileana Nachescu stakes a claim for a tactical collective intersectional feminist Eastern European identity (Nachescu, 2018 193, 197–8; see also Hendl and Nachescu, 2023) building upon Mohanty’s (2003) argument about a collective South Asian identity. Without a particular focus on CEE&E, women’s and gender studies would likely continue to collapse this part of the world into the categories of the Global North and South.

### Who should have the authority to speak about gender in CEE&E?

The *Handbook* argues that the study of gender in CEE&E has been constituted by several intense scholarly debates. These debates emerged quickly and publicly in Drakulić’s (1992) “Letter from the United States…” in which she questions the practice of generalizing across CEE&E, doubts the applicability of Western feminist theory, and criticizes what she sees as Western feminist scholars’ hubris in explaining the situation to those in CEE&E. As Hinterhuber and Fuchs (2022) explore, this first debate contained and initiated at least two other debates that then also became public and pointed: how to evaluate the feminist activism that emerged in CEE&E after 1989 and how to assess communist-era women’s organizations and policy. While the latter two have been more prominent in the first two decades of the 21st century, by 2022–3—in the face of COVID-energized right-wing anti-gender politics, the anticolonial imperatives of BLM, and the global dynamics around Russia’s full-scale invasion—the focus became the question of who has the authority to speak about gender in CEE&E.

For example, at the roundtable on gender and socialism at ECPG (2022), Zaharijević linked the first two debates, arguing that the “hierarchies” within transnational feminist scholarship is because post-socialist feminisms are “deprived” by the myth that they do not or cannot take on neoliberalism and are only doing “culture and recognition.” She further argued that these debates “turn us against each other.” As elaborated (Workshop April 21, 2023), she advocates that feminist scholars from CEE&E read more of each other’s work (as well as that by feminist scholars of CEE&E), rather than the Western feminist theorists such as Judith Butler and Nancy Fraser who have commanded so much attention and know little about “this proverbial non-region.” Ghodsee and Mrozik (2023) linked the first and third debate, arguing that there is systemic undervaluing of CEE feminist scholars, especially younger scholars and those who have a more positive understanding of communist-era women’s organizing and policy, as a result of the Cold War politics of the study of CEE&E.

Reflecting a trend more broadly within academia, other scholars have questioned the authority of Western or West-based scholars of CEE&E. In several instances in the last few years, scholars have relayed to me in confidence of having been told to keep silent in meetings because of their Western-ness, including some who have emigrated from CEE&E to escape the backlash and/or pursue their higher education. Wiedlack (2020) examines these fraught dynamics exhibited at a 2017 conference in Vienna where the organizers promoted a culture in which those presumed to be privileged (such as by Western-ness, race, gender, sexuality) were called out, but then faced accusations of missing the colonial privileges within CEE&E. In response to the 2022 call for papers for the Workshop on Gender and Transformation in CEE&E, Nachescu wrote on Facebook: “Eastern European women, as the true experts in our own histories and communities and lives, are always contributors and never editors; always one-time underpaid presenters and never series coordinators; always conference attendees and never keynote speakers; always data providers and never theorists and experts.” On Jan. 14, 2023, she tweeted, “Reading western feminists writing about Eastern Europe and honestly it’s traumatizing. Epistemic marginalization of Eastern European women masquerading as feminist solidarity.”

Tereza Hendl and Nachescu (2023) tweaked and elaborated this argument in the Spring 2023 AWSS newsletter. Beginning with some anecdotes critical of Western feminist scholars’ engagement in CEE&E over the last three decades, they issued the following evaluation:

Western feminisms produce a monolithic Eastern European woman that is white (rarely, if ever, Roma or Muslim), racist, anti-Semitic, politically conservative, enamored with consumerism, and unable to comprehend western leftist critiques of capitalism. This construct allows western “theorists” to engage in data mining; use us as case studies, othered and reduced to stereotypical caricatures; portray us as intellectually inferior, lagging behind and needing benevolent guidance to catch up with a proper western discourse, as Madina Tlostanova reminds us; hire us as cheap but diligent labor on grants and guest lectures; and erase and appropriate us in debates about our own societies, histories, feminist and resistance movements, or even sex lives… In these discussions about gender occurring in various spaces controlled by western feminists, gender is the only category of analysis, thereby promoting white (western) feminism.

They then called for scholars from the Global West to reflect on their role in this “imperialist knowledge production” and adopt “an alternative positionality” that stems from “a decolonial framework that will, among structural global inequalities, also analyze the extremely unequal power relationships between Western and Eastern European women.” Finally, they advocate a “Writing from Eastern Europe” perspective “centering and building on East European knowledges, socio-historical experiences, agency, and perspectives.”

There are several claims in these moves that I want to unpack here. The first is the “authentic insider” (Narayan, 1997) argument that only those from CEE&E have intellectual authority about this part of the world. Unsurprisingly, this is the claim with which I most struggle. I think it silences when we want more space to think more deeply in conversation with each other. It also leads us to painful internecine debates over who counts (is it only people who stay in CEE&E? what about those educated in the West and then return? what about those from privileged backgrounds within CEE&E?) without answering the question as to what to do with anti-feminist authentic insiders and movements. As Ghodsee pointed (personal communication, summer 2022), we also do not want to discount the agency of some CEE&E feminists who have by now gained their own prestige by virtue of their work. Also, I do not think we want those of us who come from globally privileged backgrounds to stop learning—which I think happens in writing, revising, and publishing—about CEE&E or the “majority world.”

The second move is both an intellectual and structural critique about the use (and abuse) of women and feminist scholars from CEE&E. Western scholars might link the intellectual basis of these arguments to feminist standpoint theorizing, but Hendl and Nachescu point to arguments made by Hana Havelková, which I see as grounded in Drakulić (1992) formative essay, which launched much intellectual reflection among Western scholars of CEE&E that Hendl and Nachescu gloss over [see Hinterhuber and Fuchs (2022) for discussion]. The structural problems are entrenched. Western scholars in more established universities and networks, especially those whose native language is English, may crowd out CEE&E feminists. Scholarly and journalist venues are not quite 0-sum, but there are limited opportunities which can only be stretched a bit here and there for discussions of gender and feminism in CEE&E, especially in the most prestigious journals, newspapers, magazines, and presses in this seemingly perpetual age of austerity. There are neoliberal “infrastructure problems” of the dominance of English-language scholarship, now reinforced by the mathematical assessment of publications for tenure at many European institutions (Ghodsee Workshop April 21, 2023).

Many of us in the West have tried to address these concerns pragmatically. The Network East–West Women, for example, strived to create more horizontal organizing—moving its leadership to Poland—while also providing needed resources to feminist scholars and activists in the region (Funk, 2022). My attempts have been the following: co-authoring chapters and articles with two junior scholars from CEE&E, including junior scholars from CEE&E as authors of half the chapters in my first edited book, co-editing the *Handbook* with two scholars with roots in the region and including scholars from CEE&E as authors in 28 out of 51 chapters, and, as one coordinator since 2008 of the Workshop on Gender and Transformation in CEE&E, bringing in as many junior (and some senior) scholars from CEE&E as possible to speak at the and sharing all the monetary resources that we had or could create; as of the last few years, we moved the Workshop mostly online and rescheduled the timing in order to facilitate the participation of CEE&E feminists. I work to follow and reference the scholarship from and about the whole of CEE&E, not just Russia, and give voice to the activists I study. I keep thinking about what else can be done—and I work within my own structural constraints as I work at a chronically underfunded minority majority urban university.

I—and Hendl and Nachescu in their essay—suggest that Ukrainians and Ukrainian feminists given us some additional insight through the concept of “Westsplaining,” which builds on the somewhat older term “mansplaining” for when men condescendingly and patronizingly explain things to women that the men could easily have seen the women were likely to know. Since Russia invaded in 2014, there have been a lot of Westerners, with little training and background in the CEE&E, let alone in Ukraine, who have explained Russia’s warmongering to the world and even to Ukrainians. As Hrytsenko (2020) pointed out, this Westsplaining tends to be gendered, most often from “[f]irst-world heterosexual educated men” who have not reflected on their privilege. The concept of Westsplaining thus helps capture both gendered and global narcissistic dynamics of those who do not think about their positionality who speak before listening, and who presume their authority matters more than others in the conversation. While the concept does not directly point to Western feminist scholars, Galyna Kotliuk (Kleve) asked us to give some reflection before we speak, especially regarding Ukraine: “I ask you to be very critical of any people’s opinions who are not from Ukraine.”

This Ukrainian perspective can then help Western feminist scholars of CEE&E to consider when to use their privilege. For example, when there are not Ukrainian or other CEE&E feminists in the conversation, we can counter de-contextualized, de-historicized, and de-gendered narratives about Russia’s war in Ukraine. We can consider speaking when we can serve as translators and amplifiers of the work by feminist scholars of the CEE&E, not just cultural imperialists, including, for example, their insights about the US’s abortion politics (Ghodsee, Workshop April 21, 2023). These kinds of logics can be useful for any of us trying to write about other places, especially those less privileged in knowledge production.

This brings me to third claim, this specifically from Hendl and Nachescu, that I want to mention: the call for Western feminist scholars to adopt a decolonial, intersectional standpoint feminist framework. This, I think, is the new debate for all of us in the gendered study of CEE&E to have, and the conversation I am trying to engage in here.

### How have the possibilities for solidarities among feminist scholars and activists across CEE&E and beyond CEE&E shifted—and why?

At the 2022 ECPG roundtable on gender and postsocialism, Katalin Fábián wondered out loud if there was a new debate over the war/invasion in Ukraine, but for the most part, this has been more a question of feminist solidarity, about the mutual support for each other around a common interest which has often been understood as a question of unity. These dynamics are documented in the special issue from the Kharkiv Center for Gender Studies on the 2022 escalation:

transnational feminists of different countries did not remain silent – they immediately condemned Russian aggression and declared their solidarity with Ukraine and their Ukrainian adherents. These were both individual statements and collective manifestos signed by hundreds of feminist scholars and activists. Looking back at the events of last Spring, we can say that a truly mass feminist anti-militarist mobilization took place during these days. However, not everything is so simple and cloudless within this international feminist mobilization and solidarity… (Zherebkina et al., 2022).

In reflecting on the related online meeting on “Transnational feminist solidarity with Ukrainian feminists” organized by Judith Butler, Sabine Hark, and Irina Zherebkina on May 9, 2022, the editors pointed to three sets of disagreements: “(1) disagreements due to the EastWest divide, fixed by a number of authors representing central and eastern Europe; (2) disagreements between (a) feminist ethics of non-violence and (b) feminist arguments in defense of women’s discourses and practices of violence and revenge; (3) disagreements between transnationalism and nationalism and some others.” The first of these speaks to the debate about who can study gender in CEE&E discussed above, but the latter two have involved little scholarly disagreements to date [for a critique of the intellectual arguments, see Hendl (2022) in the special issue].

Instead, in this time of war—with very real impact in terms of Ukrainian need for Western societies’ support—these latter two were more about meaningful and measurable commitments to Ukrainian feminists. As Maryna Shevtsova pointed out at the ECPG (2022) roundtable on Ukraine, the March 14 “Feminist Resistance Against War Manifesto”^8^ issued by European feminists condemned the invasion of Ukraine, but also called for demilitarization without prioritizing the demilitarization of Russia (which she called a “terrorist state”); she also noted that the manifesto was crafted without discussion with Ukrainian feminists. Audience members from CEE at the roundtable stated that they had never felt more Central-Eastern European—as Western Europeans, especially those in the Left, do not have the same deep visceral fear of Russian aggression and invasion rooted in collective historical memory. Mieke Verloo, a Dutch political scientist, decried the uninformed manifesto, explaining pacifism must work differently when “it’s not just a war, but an invasion” and “we need new gender theories about war when anti-gender politics have turned into violence.”^9^ The same day as the ECPG panel—July 7—Ukrainian feminists released their own manifesto, criticizing the “abstract pacifism” of the first manifesto:^10^ “[w]e, feminists from Ukraine, call on feminists around the world to stand in solidarity with the resistance movement of the Ukrainian people against the predatory, imperialist war unleashed by the Russian Federation.” Almost a year later (February 2023), Western anti-war activists doubled down, again without the voices and viewpoints from Ukraine or Ukrainian feminists. Prominent self-identified feminist Alice Schwarzer (along with Left MP Sahra Wagenknecht) issued another manifesto (and organized a large protest) calling for Germany to stop providing weapons and start pushing harder for peace.^11^ While German anti-war activists have their own reasons, there is also compelling evidence that the Kremlin has been working on supporting this movement since September 2022; and there was at least one person close to Wagenknecht who was in contact with Russian officials at that time (Belton et al., 2023). US-based Code Pink has made similar efforts, with one co-founder (Medea Benjamin, with no background in CEE&E) claiming to support Ukrainian citizens and condemning the war, but also the US for “fomenting the war” with NATO expansion^12^ and the other (Jodie Evans) implicated as “part of a lavishly funded influence campaign that defends China” and, it seems by her statements, Russia (Hvistendahl et al., 2023).

Another dimension of these questions of solidarity relate to the way that Western feminists framed their assistance. Inna Iryskina (2022) points to the ways in which Western journalists and feminists homed in on the concerns facing transgender people trying to leave Ukraine as if they were “poor passive victims” and the Westerners were “saviors,” without acknowledging the activism and progress on LGBTQ issues and the desire of many to stay and resist—or our own failures on transgender rights. At the Kleve conference, Galyna Kotliuk argued that Western manifestos seemed to reflect a “White Savior complex” with Ukrainians are “Orientalized as blue-eyed savages,” suggesting something similar to the concept of peripheral whiteness.

There was an outright schism between Ukrainian feminists and Russian feminists over the full-scale war. Several Ukrainian feminists took a political stance to not attend events which included Russia-based scholars. To my knowledge, this began with the AWSS (hybrid) conference hosted by The Melikian Center: Russian, Eurasian & East European Studies, Arizona State University at the end of March 2022.^13^ Rectors of virtually all universities in Russia had signed two statements in support of the war and Putin’s historically inaccurate justifications.^14^ Several scholars with heritage from Ukraine wrote AWSS asking that Russia-based scholars be excluded from the conference. AWSS refused, stating that inclusion/exclusion should be based on opposition/support for the war.^15^ A similar discussion and decision by organizers apparently also happened at the online “Transnational Feminist Solidarity with Ukrainian Feminists” (Zherebkina et al., 2022). In other situations, feminist Ukrainian scholars have refused to share space with discussion of Russian anti-war activism or Russian losses because it suggests “moral equivalence.”

In 2023, the schism remained, even for the (Russian) Feminist Anti-War Resistance whose stance against the war was quick and unequivocal.^16^ According to one spokesperson, Ella Rossman (2023), “it’s understandable” that organizations in Ukraine “do not want to work with us in this situation;” instead, they have tried to amplify the voices of Ukrainians talking about the war as well as assist Ukrainians who are kidnapped or forced to flee into in Russia find a way out. On the other side, according to Vanya Solevey (Kleve), self-identified feminist group WomenNation and the self-proclaimed feminist Bella Rapaport have helped with Russia’s propaganda efforts. As a Jewish out lesbian who has stayed in Russia, Rapaport’s positionality is complicated, but her social media comments have at least been “tone deaf” toward the war, and Ukrainian feminists (led by Dafna Rachok) got her disinvited to the 2023 conference of the British Association for Slavonic and East European Studies (Alexandra Novitskaya, personal communication, May 24, 2023).

The schism was not just about the full-scale war but a history of the power differentials that have been unmarked and unseen by most feminists in Russia and most Western feminists. As explained by Hrytsenko (2022b), feminism in Ukraine in many ways came from the West, filtered through Russian translations and online venues, like the Russian-based platform *feministki*. She writes:

Ukrainian feminists, who are located in the common information field, partially broadcast the Russian agenda (e.g., announcements of events and lectures) on their social media pages, but it is always an unequal and unfair exploitation of labor in the media space and never mutual support. Russian feminists do not post Ukrainian announcements, they are not interested in the stories relevant to Ukrainian feminists, they do not sympathize with difficulties faced by the Ukrainian feminist community, nor do they rejoice in its success. Occasionally though, Russian feminists react harshly to requests for support and solidarity.

She calls the war, which started for Ukrainians in 2014, “a fault line between Ukrainian and Russian feminism” (Hrytsenko, 2022a). In an analysis of a 2014 discussion on *feministki*, she illustrates how strong the anti-Ukrainian and pro-Imperial Russia sentiments were, including from prominent Russian feminists such as Natalia Bitten, and they were left unquestioned by the Russia-based moderators.^17^

Over the last decade and a half, as those focused on gender justice consider feminisms in new ways in CEE&E, there have been many schisms. More than just debates, activists have been polarized and divided by discussion about whether lesbianism is part of the feminist agenda, over prostitution vs. sex work (and the Swedish model), and about trans issues, with strong TERF (trans-exclusionary radical feminist) stances by some. Hrytsenko (2022b) points to Russian feminism for this too, arguing that the anti-prostitution and transphobic stances that had been embraced by Ukrainian feminists were adopted uncritically from the West, often through “Russian dissemination.”

But, on the war, feminisms in CEE&E (other than in Russia) have been mostly united in pragmatic solidarity. Polish feminists rallied to provide tremendous support, including helping some Ukrainian refugees get illegal abortions, and Germans who helped bring the medicines in the first year of war when supply chains got broken. Simona Fojtova (ASEEES 2022), speaking of Czech support for Ukrainian refugees, suggests this should not be considered grassroots humanitarianism, but “mutual aid,” radical collective care that is anti-authoritarian and participatory. For example, “grandmothers without borders,” a group in action since the 2015 Syrian refugee crisis, opened a “women’s cafe” for Ukrainians refugees in March 2022.

Since 2022, activists and scholars have been suggesting that the best way forward is to consider new ways of being in solidarity, but this has not resolved the conflict. Helen Petrovsky and Sabine Hark, in the Kharkiv special issue, propose a definition of solidarity that relies “on disagreement, questioning and contestation,” but having shared interests (Zherebkina et al., 2022, 6). At the ECPG (2022) panel on gender and postcommunism, there were calls for some kind of solidarity. Belgrade-based Zaharijević explained:

Solidarity should not be neglected but…, as Gregor and Grzebalska (2016) once underlined, redefined so that it acknowledges positionality… This positionality would entail where we actually were before the onset of neoliberalism, where we were after the fall of Iron Curtain, and where we are now, where there is still Velvet Curtain that falls differently on different regions of the peripheral Europe. This positionality could encourage our coalitional potential, without pushing us into mythologisation that, in the end, serves us little, but is greatly fueling what is so wrongly called a culture war.

For Zaharijević, what is now needed is “many more border-crossings.” However, there were some limits. US-born Ghodsee, zooming from Germany because she had caught COVID, called for “strategic homogenization,” a riff she said on Gayatri Spivak’s “strategic essentialism,” to counter the many divisions and failures within the Left, while recognizing intersectionality, positionality, hierarchies. Ghodsee’s take seemed to me to be similar to Zaharijević’s in its call for a coalitional solidarity, but it did not land well in the room. At the panel, Hungarian-born, US-based Fábián explicitly rejected this idea of homogeneity. Given the historical context—but also perhaps the specificity of this roundtable being in the same room as and directly following the panel on the war in Ukraine, with many of the same attendees—any kind of idea that included homogeneity was too colonial.

In 2023, there have been a few signs that new kinds of solidarity can be built as feminist activist-scholars seek constructive dialog around Russia’s invasion of Ukraine through thoughtfulness and unexpected intersectionality. For example, at the Kleve conference, Manuela Scheuermann importantly pondered over the Ukrainian feminist “visions of peace for after the war, given that Ukrainian feminists are asking for more weapons,” which is very different from the pacifist feminism of the German feminist manifestos. Olena Strelnyk respectfully responded, “Ukrainian feminists are not rejecting pacifism, they are showing the failure of the post WWII peace-building process.” At the Workshop (Feb. 24, 2023) event on the first anniversary of the war, feminist CEO of Project Kesher Karyn Grossman Gershon—in conversation with Oksana Kis and Olena Nikolayenko—illustrated the way that claiming space for Jewish feminism in Ukraine is a transformative act, undermining Putin’s claims that the war is somehow about de-nazifying Ukraine. These conversations and events suggest a way forward for feminist scholars of CEE&E to theorize better around gender and war in the 21st century in ways that take not just gender, but colonialism in all its forms, seriously, and perhaps finding ways to advocate for feminist and just peacebuilding in these historical bloodlands and borderlands.

## Implications for gender studies

I will not conclude as the war remains devastatingly on-going. My intention was to suggest a framework for thinking about the questions that scholars of gender in CEE&E might want to reckon with based conversations I have observed since February 2022. In sum, I think that there is still much to be gained from studying gender in CEE&E as a field in 2023 if we incorporate intersectional and decolonial lenses and especially if we can keep pushing ourselves through thoughtful scholarly debates. Schisms may be necessary growing pains, especially in this current moment of Russia’s war on Ukraine, but I urge us to keep trying to be in careful dialog with each other while we keep reflecting on the gendered, global, and other power dynamics at play as this may allow solidarity based on coalitional politics.

This intersectional decolonial exploration privileging Ukraine points to questions for gender studies scholars as a whole. Might we need to think more about the ways that postcolonial and decolonial thinking has conflated race with colonialism? Is it useful to use concepts such as peripheral whiteness or Orientalization in such cases or might we need to wrestle more with the intersectional complex of subordination and dominance when privileged by race and subordinated by colonialism? Similarly, might we need to grapple more with the complexities of socialism, whose legacies include some empowerment along gendered lines, but was imbricated with Soviet colonialism? Might we need to contend more with differences between epistemic imperialism, such as that by Western (or even Russian) feminists, and colonial conquest? For those of us from the West, might the Ukrainian feminist concept of Westplaining help give us pause and insight before we speak or imagine feminist solidarities? As feminist scholars of the Global South led the charge toward feminist decolonial approaches, feminist scholars of CEE&E can and should reveal the dynamics of this different kind of colonial dynamics.

## Data availability statement

The original contributions presented in the study are included in the article/supplementary material, further inquiries can be directed to the corresponding author.

## Author contributions

The author confirms being the sole contributor of this work and has approved it for publication.

## Footnotes

^1^In the last paragraph of this introduction, I list the various exchanges and academic events that I use as evidence and the names or acronyms I use to reference them. In this article, I use only informal in-text reference to scholars, the event, and the year as they are from unscripted participation, unpublished manuscripts, or social media.

^2^As the 2022 conference was in Slovenia, there were many panels related to CEE&E, but these were the two most relevant panels: “The war in Ukraine: an open discussion for the ECPG community “and “Postsocialism and gender equality,” https://ecpr.eu/Events/157.

^3^I refer to these panels especially: “Studying Ukrainian and Russian Domestic Politics in Times of War and Peace,” “Gender, Sexuality, and Violence in the Context of the 2022 Escalation of Russia’s War on Ukraine,” “The Gender of War: Central and Eastern European Anti-Gender Crusades,” a roundtable on the *Routledge Handbook of Gender in Central-Eastern Europe and Eurasia*, and “Gender, Sexuality, and Violence in the Context of the 2022 Escalation of Russia’s War on Ukraine (online),” https://www.aseees.org/sites/default/files/downloads/2022%20Convention%20Program%20FINAL%20to%20post%20sm.pdf.

^4^For details on the workshop and the events, see https://ceeegender.commons.gc.cuny.edu/.

^5^ https://genderindetail.org.ua/

^6^“FRIAS supports Ukrainian researchers,” https://www.frias.uni-freiburg.de/en/frias-supports-ukrainian-researchers.

^7^“Historical Fakes in Russian Media during the First Phase of the Russian Aggression Against Ukraine (2014–2016),” Jan. 31, 2023, Columbia University.

^8^See https://www.greenleft.org.au/content/right-resist-feminist-manifesto-ukraine.

^9^See also Feminist Fightback. 2022. “Why we need feminist solidarity with Ukraine,” May 26, http://www.feministfightback.org.uk/why-we-need-feminist-solidarity-with-ukraine/.

^10^The Feminist Initiative Group. 2022. ‘The right to resist’: A feminist manifesto from Ukraine,” July 13, https://www.greenleft.org.au/content/right-resist-feminist-manifesto-ukraine

^11^“Wagenknecht und Schwarzer warnen vor Ausweitung des Krieges” [Wagenknecht and Schwarzer warn against escalation of the war]. 2023, *NDR*. Feb. 11, https://www.ndr.de/kultur/Wagenknecht-und-Schwarzer-warnen-vor-Ausweitung-des-Krieges,wagenknecht300.html

^12^Video: “War in Ukraine: Making Sense of a Senseless Conflict,” https://www.codepink.org/ukraine. https://www.nytimes.com/2023/08/05/world/europe/neville-roy-singham-china-propaganda.html

^13^ https://awsshome.org/about/conferences/

^14^Hop. 2022. “Russian rectors support war in Ukraine,” DUB, Mar. 8, https://dub.uu.nl/en/news/russian-rectors-support-war-ukraine

^15^“AWSS’s biennial conference welcomes all students and scholars who oppose Russia’s war on Ukraine. Anyone who supports Russia’s war or justifying it in any way, will not be welcome.” https://awsshome.org/wp-content/uploads/2022/03/AWSS-Conference-Schedule_update_2022-03-18.pdf. Marta Havryshko, who was one of those that signed the statement, told me that they were waiting for the Russians to take a clear stand against the war, which they had not yet (Association for the Study of Nationalities, May 2023).

^16^Feminist Anti-War Resistance. 2022. “Russia’s Feminists Are in the Streets Protesting Putin’s War,” *Jacobin*, Feb. 27, 2022, https://jacobin.com/2022/02/russian-feminist-antiwar-resistance-ukraine-putin.

^17^At Kleve in 2023, Russian feminist Vanya Solovey foregrounded Ukrainian feminism: “Ukrainian feminism has influenced Russian femimisms, Russian feminists have copied Ukrainian initatives, followed Ukrainian examples at some crucial points,” most notably “the very first online community *feministki* (2005) was modeled on *feminismua* (founded 2004)” and #Iamnotafraid online flashmob was started by Ukrainian feminists.”
