# Corrigendum: How Russia's war in Ukraine can change gender studies

**DOI:** 10.3389/fsoc.2024.1386270

**Published:** 2024-03-13

**Authors:** Janet Elise Johnson

**Affiliations:** Brooklyn College (CUNY), Brooklyn, NY, United States

**Keywords:** intersectionality, decolonization, solidarity, Ukraine, postsocialisms, Russia, gender studies

In the published article, the name of one of the authors was incorrectly spelled in the reference for Schurko, T., and Suchland, J. (2022). Postcoloniality in Central-Eastern Europe and Eurasia. Hand, 71–79. as Schurko. It should be Shchurko.

In the published article, the name of one of the authors, Ileana Nachsecu, was incorrectly spelled in the text. A correction has been made to **Raising questions and some tentative thoughts**, *Does CEE&E (still) constitute a meaningful geopolitical region for understanding gender?*, Paragraph 9. This sentence previously stated:

“For example, Ileana Nachsecu stakes a claim for a tactical collective intersectional feminist Eastern European identity (Nachescu, 2018 193, 197–8; see also Hendl and Nachescu, 2023) building upon Mohanty's (2003) argument about a collective South Asian identity.”

The corrected sentence appears below:

“For example, Ileana Nachescu stakes a claim for a tactical collective intersectional feminist Eastern European identity (Nachescu, 2018 193, 197–8; see also Hendl and Nachescu, 2023) building upon Mohanty's (2003) argument about a collective South Asian identity.”

The author apologizes for these errors and states that they do not change the scientific conclusions of the article in any way. The original article has been updated.

